# Methods for Manipulating *Cryptococcus* Spores

**DOI:** 10.3390/jof8010004

**Published:** 2021-12-22

**Authors:** Anna B. Frerichs, Mingwei Huang, Sébastien C. Ortiz, Christina M. Hull

**Affiliations:** 1 Department of Biomolecular Chemistry, School of Medicine and Public Health, Madison, WI 53706, USA; afrerichs@wisc.edu (A.B.F.); huangmingwei05@gmail.com (M.H.); sortiz2@wisc.edu (S.C.O.); 2 Department of Medical Microbiology & Immunology, School of Medicine and Public Health, University of Wisconsin-Madison, Madison, WI 53706, USA

**Keywords:** human fungal pathogen, *Cryptococcus*, spore purification, density gradient centrifugation, germination assay, cryptococcosis, basidiospores

## Abstract

Spores are essential for the long-term survival of many diverse organisms, due to their roles in reproduction and stress resistance. In the environmental human fungal pathogen, *Cryptococcus*, basidiospores are robust cells with the ability to cause disease in animal models of infection. Here we describe methods for producing and purifying *Cryptococcus* basidiospores in quantities sufficient for large-scale analyses. The production of high numbers of pure spores has facilitated the development of new assays, including quantitative germination assays, and enabled transcriptomic, proteomic, and virulence studies, leading to discoveries of behaviors and properties unique to spores and spore-mediated disease.

## 1. Introduction

Sporulation is vital for the survival of many diverse organisms as a mechanism to survive until suitable conditions for vegetative growth are met [1,2,3]. Fungi are particularly efficient at both spore production and dispersal, and fungal geneticists have capitalized on the connection between sexual development and spore formation to carry out classical genetics in many fungal model systems (e.g., *Saccharomyces cerevisiae*, *Neurospora crassa*, and *Aspergillus nidulans*). The manipulable sexual cycles of these organisms coupled with the ability to isolate recombinant spores led to powerful tools for identifying, characterizing, and manipulating genes in these organisms [4,5,6]. These developments greatly progressed the understanding of fundamental fungal biology and other eukaryotic processes.

Among the human fungal pathogens, the opportunity to take advantage of classical genetics has been more limited for a variety of reasons; however, the environmental yeast *Cryptococcus* is a notable exception. Since *Cryptococcus* sexual development was discovered in the 1970s, the use of spores to definitively link genotypes to phenotypes has been a mainstay of the system [7]. It could be argued that development of *Cryptococcus* into the model human fungal pathogen that it is today resulted in large part from the fact that basidiospores (known as spores hereafter) could be manipulated for analysis using classical genetic approaches [8]. This facilitated the development of other molecular-genetic tools, ultimately creating a system for the study of fungal pathogenesis with broad utility.

The production of spores by *Cryptococcus* occurs via sexual development between either haploid yeast of opposite mating types (**a** and α) or of a single mating type (α cells alone). Under appropriate conditions, yeast strains fuse with a partner or undergo endoduplication and then initiate filamentous growth. In response to unknown signals, the terminal filament cells form microscopic fruiting bodies (basidia) in which meiosis and repeated mitoses occur. The mixed haploid mitotic products are then packaged, and spores bud onto the surface of each basidium in four chains [8,9].

Because *Cryptococcus* spores are not contained in an ascus, they can be isolated directly from basidia and analyzed on a per chain, per basidium, or population level (random spore analysis) using microdissection of individual spores [10]. While microdissection is invaluable as a genetic tool, it is impractical for use in the generation of large numbers of spores for more comprehensive analyses of spore properties. Anecdotally, many attempts were made to isolate *Cryptococcus* spores in larger numbers over the decades, but methods used successfully in other systems were not adequate to yield large numbers of pure *Cryptococcus* spores, thus preventing studies to determine their physical, biochemical, molecular, and virulence properties. Therefore, the vast majority of studies of *Cryptococcus* biology have focused on the yeast form. It was not until over 50 years after the initial report of spores in crosses that sufficient numbers of spores were isolated for fundamental studies. This breakthrough facilitated the discoveries that *Cryptococcus* spores are more resistant to stress conditions than yeast, they are infectious particles in mammalian disease, they are more likely to cause central nervous system disease than yeast, and they are a potential source of new antifungal drug targets [11,12,13,14].

The methods presented here represent the approaches that have been taken to evaluate *Cryptococcus* spores from how to generate and purify them in large quantities to how to use quantitative methods to evaluate population-level behaviors. They are intended to facilitate the use and study of spores to discover fundamental fungal spore biology, properties of fungal infectious particles, and mechanisms of pathogenesis of one of the most common and deadly invasive fungal pathogens.

## 2. Materials and Methods

Success in spore purifications is dependent on two primary factors: generating large numbers of spores per basidium during sexual development and maximizing spore sedimentation without yeast contamination during gradient purification. Variables to consider for production and purification purposes are species/strain background, environmental conditions to induce sexual development, length of time of development, and percent of Percoll in purification gradients.

### 2.1. Producing Spores from Cryptococcus

The most efficient mechanism by which to produce *Cryptococcus* spores for purification is to carry out **a** x α crosses with the serotype D, congenic type strains JEC20 and JEC21 on V8 pH 7 agar plates at 22 °C for 5 days in the dark. These crosses produce large numbers of spores that efficiently sediment in Percoll^®^ gradients, leaving all other cell types behind, including yeast. This approach reliably yields >99% pure spores with well over 95% germination efficiency on rich growth media.

#### 2.1.1. Strain Selection

JEC20 x JEC21 [15,16]: spores from crosses between the congenic laboratory type strains JEC20 and JEC21 are relatively easy to purify because they produce large quantities of spores, and the yeast from these crosses do not co-sediment with spores during centrifugation, making yeast impurities generally low (<1%). The following purification protocol has been optimized for this strain pair. The JEC20 and JEC21 yeast grow poorly at 37 °C and do not cause disease in C57BL/6 mice in an intranasal model of infection; however, high concentrations of spores from JEC20 x JEC21 crosses (1 × 10^7^ cells/mouse) can cause fatal disease. Both yeast and spores of this background can cause disease in mice when injected via tail vein (1 × 10^6^ cells/mouse), and do so with the same kinetics [13].

B3502 x B3501 [17]: spores from crosses between the non-congenic serotype D strains B3502 and B3501 (F1 siblings) make fewer spores than JEC20 x JEC21, and yeast contamination is common due to yeast co-sedimentation in the gradient. The yeast of these strains do not cause disease in C57BL/6 mice in an intranasal model of infection (2.5 × 10^5^ cells/mouse); however, spores reliably cause disease in mice by 75 days post infection (2.5 × 10^5^ cells/mouse) [13].

KN99**a** x KN99α [18]: KN99**a** and KN99α are a congenic strain pair derived from the virulent serotype A type strain H99. Crosses from these strains should be performed on pH 5 V8 plates, but still produce fewer spores than JEC20 x JEC21 and are more challenging to purify. Large-scale gradient centrifugation can result in high levels of yeast co-sedimentation. Both the yeast and spores of these strains cause disease in C57BL/6 mice via inhalation models with 1 × 10^5^ cells/mouse within 30 days of infection [19].

BT63 x H99 [20]: serotype A strain BT63 is a Botswanan strain that when crossed with H99 produces copious quantities of spores that can be purified by gradient centrifugation but experience higher yeast contamination than spores from JEC20 x JEC21. Notably, only ~50% of the progeny are viable to germinate due to high genetic diversity between the strains. BT63 and H99 strains cause disease as both yeast and spores in C57BL/6 mice when intranasally infected with 1 × 10^5^ cells/mouse. Spore infected mice display higher fungal burden in the brain but both cell types cause death with the same kinetics [13].

*Cryptococcus gattii*: while many *Cryptococcus gattii* strains have been shown to produce spores in quantities likely sufficient for purification, there are currently no protocols in place for gradient purification of these spores. *C. gattii* can cause disease in healthy individuals so any work with large quantities of *C. gattii* spores should be carried out under BSL3 and ABSL3 containment [21].

#### 2.1.2. Spore Production

The process of spore production and isolation takes a minimum of 9 days for JEC20 x JEC21 (and longer if using other strains and conditions): 2 days to grow from −80 °C freezer stocks, 2 days to grow a lawn of yeast, and at least 5 days for sufficient sporulation to occur (additionally, V8 plates must be aged at least 14 days before they will be fully effective for robust sexual development).

**Figure 1:**
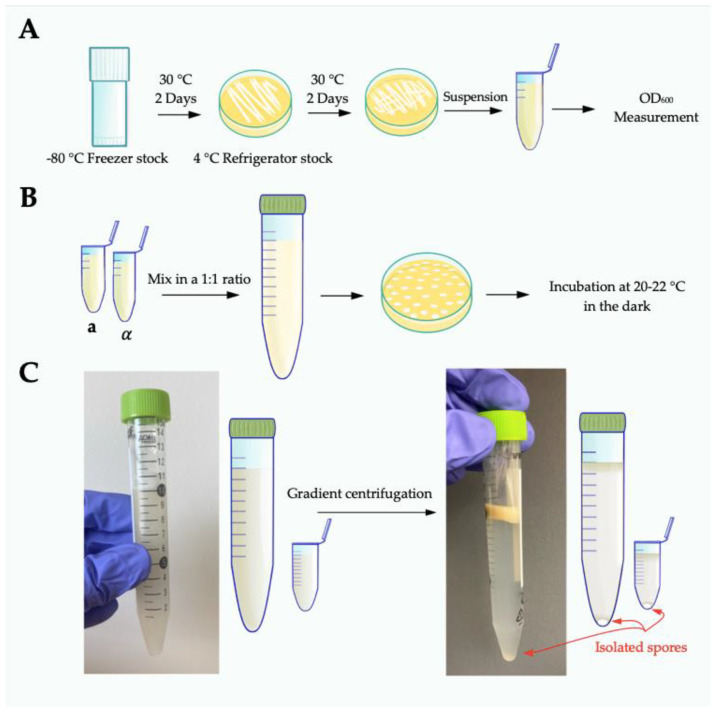
Illustrations of spore purification using JEC20 x JEC21 crosses. (**A**) Recover strains from the −80 °C freezer and plate on YPD agar. Grow for 2 days at 30 °C. Transfer sufficient cells and streak to a new YPD plate, and grow for 2 days at 30 °C. Suspend cells in 1 mL PBS for OD_600_ measurement. (**B**) Mix strains in a 1:1 ratio in 10 mL PBS to a final total OD_600_ equal to 2.0. Plate cells to V8 plates in 10 µL spots using a multichannel pipette. Incubate at 20–22 °C in the dark with the agar side down for 5–7 days. (**C**) Scrape the total mass of crosses and resuspend in 75% Percoll^®^. Mix thoroughly to homogeneity by vigorous vortexing (left panel). Centrifuge at 4 °C at 4000 RPM/21,410× *g* in a swinging bucket rotor for 25 min to generate a density gradient. Spores will sediment to the bottom of the tubes, all other cell types will be at the top of the gradient. In a successful purification gradient, there will be a clearly visible separation between spores and all other cell types (right panel).

(1) Two weeks before crossing, make large (15 cm) V8 juice agar plates: 5% V8 juice (Campbell Soup Co., Camden, NJ, USA), 2.5% Bacteriological agar (VWR International, Radnor, PA, USA, ref. no. IB49173), 0.5 g/L potassium dihydrogen phosphate, pH 7.0 (pH with 5 M potassium hydroxide). Pour plates when V8 medium is cooled to ~45 °C to minimize condensation. Allow plates to cool in short stacks (1–5 plates) at room temperature overnight. After cooling, age plates unbagged at room temperature away from direct sunlight for 14 days. After 14 days, put plates in plastic bags to prevent desiccation. Store at room temperature in the dark.
(2) Four days before crossing, recover strains from −80 °C freezer stocks by streaking to rich growth medium (10 cm YPD agar) and grow for 2 days at 30 °C. Re-streak sufficient cells (a mass the size of a match head) to at least half of a new 10 cm YPD plate to create a lawn. Grow for 2 days at 30 °C (Figure 1A).
(3) For each strain, prepare cells by scraping up yeast from half of a 10 cm YPD plate and resuspending in 1 mL 1× phosphate buffered saline (PBS). Determine the OD_600_ for each resuspended strain stock. Mix strains in a 1:1 ratio in 10 mL 1× PBS to a final total OD_600_ equal to 2.0 (i.e., mix 1 OD_600_ unit JEC20 + 1 OD_600_ unit JEC21 and bring to a final volume of 10 mL).
(4) Plate cells to 15 cm V8 plates in 10 µL spots. Maximize the distance between spots on each plate, by plating with every other tip on a multichannel pipette. Each plate can hold up to 48 spots (Figure 1B).
(5) Let spots soak into agar before moving; incubate at 20–22 °C in the dark with the agar side down for 5 days.

Tips for success: this protocol works reliably well for crosses between JEC20 and JEC21 and generates positive outcomes for B3502 x B3501 crosses. Use of other strains, such as H99 x BT63 and KN99**a** x KN99α can be further optimized from this base protocol. For all serotype A strains, use V8 plates at pH 5.0. Seasonality and regionality of V8 juices may affect sexual development and, therefore, spore production, but sufficient quantities of spores can generally be obtained despite the fluctuation; other strains may be more sensitive to these differences and require more troubleshooting.

Ensure that V8 plates are properly sterilized, poured, and aged. Different Petri dish brands can yield inconsistent spore production. A reliable brand is Fisher Scientific (Hampton, NH, USA), 150 × 15 mm (cat. no. FB0875714). V8 media should be autoclaved long enough (60 min at 250 °C with slow exhaust) to prevent embedded contamination which can be a serious problem during storage and use. V8 medium should be about half a centimeter thick in each plate to prevent over desiccation. Plates induce spore production better after at least a two-week incubation period, and plates well over one year of age that are still in good condition (even, hydrated, sterile agar) have been used with success. The aging period is not associated with plate dryness; attempts to dry plates faster in tissue culture hoods did not replicate aging.

Yeast freezer stock plates can be stored at 4 °C for up to 4 weeks. Spore sample purity decreases dramatically after parental yeast plates reach 4 weeks of age. When setting up **a** x α crosses, cell concentration is critical, presumably for optimal pheromone signaling and fusion between cells of opposite mating types. A total OD_600_ of 2.0 is optimal for JEC20 x JEC21; however, this concentration may be further optimized for other strain pairs as needed.

Once V8 plates are spotted, allow the spots to dry briefly before moving them. For challenging strains, spotting with lower volumes and wider spacing may increase spore production. Lower humidity incubation is generally better than higher humidity; a light-limited cabinet or drawer is generally sufficient for light restriction. For JEC20 x JEC21 crosses, spore yields increase over the first five days of incubation and start to drop after 7 days. For other strains, following sporulation microscopically is recommended to determine optimal incubation times.

### 2.2. Spore Purification Using Percoll Density Gradient Centrifugation

In the 1960s, biologists capitalized on the production of industrial colloidal silica (a.k.a. Ludox^®^) to develop gradient centrifugation approaches for the recovery of live microbes from environmental samples [22]. Ludox^®^ is still used today to purify entomopathogenic algae from host insect larvae extracts [23]. Based on this foundation, we developed a purification protocol to isolate spores from sexually developing populations of *Cryptococcus* using colloidal silica nanoparticles coated in polyvinylpyrrolidone (a.k.a. Percoll^®^). Percoll^®^ generates self-forming gradients during centrifugation that are non-toxic, metabolically inert, and easily washed away from purified spores [11]. Percoll^®^ gradients can be scaled for the number of spores needed for any given experiment and are presented here as “large scale” and “small scale” purification protocols.

#### 2.2.1. Large Scale Purification (Yield ~5 × 10^7^ Spores/10 Plates)

(1) Evaluate basidia on V8 plates for adequate spore production using a microscope. Spores are generally visible around the edge of each growth spot and can be seen in chains off the basidia at 200× magnification.
(2) Prepare one 15 mL conical tube (VWR International, Radnor, PA, USA, ref no. 89039-668) per sample. One 15 mL should accommodate crosses from three to ten large (15 cm) V8 plates. Add 10 mL 75% Percoll^®^ (GE Health Care, Chicago, IL, USA, ref no. 17-0891-01; 100% Percoll^®^ diluted to 75% with 1× phosphate buffered saline-PBS) to each tube and place on ice. Use a cell scraper (Fisher Scientific, Hampton, NH, USA, ref no. 353086) to harvest the total mass of all spots off each V8 plate and resuspend in the Percoll^®^. Mix thoroughly by vortexing vigorously until the cell mass is homogenous throughout the Percoll^®^. It is imperative to fully homogenize the solution, as even small clumps of cells will sediment improperly during centrifugation, leading to impurities in the final spore preparations. If necessary, clumps of cells can be physically disrupted using a sterile plastic pipette tip. Keep tubes on ice throughout the harvesting process.
(3) Centrifuge at 4 °C, at 2110× *g*, in a swinging bucket rotor for 25 min to generate a density gradient. Spores will sediment to the bottom of the tubes while all other cell types will remain at or near the top of the gradient. There should be a clear separation between spores and all other cell types (Figure 1C). If there is visible sedimentation of cells from the top of the gradient through and down to the bottom of the tube, the spores will likely not be pure.
(4) Carefully remove tubes from the centrifuge and place on ice; do not disrupt the gradient.
(5) Collect spores from the bottom of the density gradient:
  - Gently secure the 15 mL conical tube to a ring stand, maintaining clear access to the bottom of the tube.
  - Sterilize the outside of the 15 mL conical tube with 70% ethanol and dry. Remove the cap to allow the tube to vent during the next step. Venting is critical to this process; failure to do so can create a vacuum and result in sample loss.
  - Use a 21-gauge needle (Fisher Scientific, Hampton, NH, USA, ref no. 1484092) to **carefully and gently** drill a hole in the bottom of the conical tube. It is imperative that the tube be stabilized in a manner that precludes a needle hazard. Keep both hands away from the trajectory of the needle to avoid a needle stick injury. Discard needles into a biohazard sharps container; do not recap contaminated needles!
  - Immediately collect the first 200 µL that drip out in a sterile 1.5 mL microfuge tube (5–7 drops total).
(6) Fill the microfuge tube to 1.5 mL with 1× PBS, vigorously pipette up and down to disperse the spores. Vortex to ensure spores are evenly suspended in solution. Centrifuge for 5 min at 2000 RPM (~370× *g*). Discard the top 1.3 mL of PBS, careful not to disturb the spores at the bottom. There may not be a visible pellet at this point.
(7) Repeat step 6.
(8) Carry out a final wash with 1.5 mL PBS and centrifuge for 1 min at maximum speed (13,200 RPM/16,100× *g*) to pellet spores. Discard supernatant and resuspend spore pellet in 300–700 µL PBS. Volume depends on anticipated spore yield based on visualization of spore pellet and number of V8 plates used to generate the sample. For JEC20 x JEC21 crosses after 5 days of development on V8 pH 7 agar, spore yields should be ~5 × 10^6^ spores per V8 plate.
(9) Count spores using a hemocytometer to determine final concentration and level of purity. Spores can be stored at 4 °C but begin to adhere to each other the longer they are stored.

Safety considerations: recovering spores by inserting a needle into the bottom of the 15 mL conical tube could result in needle stick injuries if proper precautions are not taken. Extra care must be taken to prevent the needle from slipping off the tube surface as it is punctured and to always keep hands and fingers out of the way. Both 21- and 23-gauge needles have been used according to user preference to generate the minimum amount of pressure necessary to drill into the tube. Note: the pellet drips out quickly, usually 5–7 drops are sufficient to recover all of the spores. To empty the conical tube, allow the remaining Percoll^®^ to drain into a beaker containing 50% bleach. Alternatively, spores can be collected from the bottom of the tube by inserting a drawn-out glass pipet from the top of the gradient and drawing up the spore pellet in ~200 µL. Extreme care must be taken with this method to avoid contaminating the spores with cells from the top of the gradient.

Tips for success: the low speed centrifuge steps are to remove the Percoll^®^, higher speeds will cause the Percoll^®^ to pellet with the spores. After each centrifugation, homogenize the spore pellet thoroughly by pipetting up and down and vortexing (the spores tend to clump). If clumping is an issue for further experiments, 0.01% Triton X-100 can be added to each wash step to prevent spores from sticking together without affecting the viability of the spores.

Crosses from some strain pairs adhere to the V8 medium more than others, which makes it more difficult to scrape crosses from the plate for spore purification. In this case, cell lifters (Corning, Corning, NY, USA, ref no. 3008) can be used more effectively due to their higher rigidity. Care must be taken to scrape off as little of the V8 agar as possible because the nutrients in the medium could potentially trigger spore germination during purification.

#### 2.2.2. Small Scale Purification (Yield ~1.5 × 10^6^ Spores Total Per Plate)

(1) Mix strains of opposite mating types together in a 1:1 ratio (OD_600_ = 2.0).
(2) Pipet 30–40 10 µL spots on one 15 cm V8 pH 7.0 agar and incubate at 20–22 °C in the dark with the agar side down for 5–7 days.
(3) Prepare one 1.5 mL microfuge tube with 1 mL 75% Percoll^®^ and place on ice.
(4) Use a cell scraper to scrape the total mass of cells and resuspend in the 75% Percoll^®^. Resuspend thoroughly by vigorous vortexing until the cell mass appears homogenous throughout. If any clumps are still visible, pipette up and down to remove them. Keep tubes on ice throughout harvest of spots.
(5) Centrifuge at 4 °C at 2000× *g* for 25 min to generate a density gradient. Spores will sediment to the bottom of the tubes. All other cell types will remain at the top of the gradient. There will be a clear separation between spores and other cells. If there is visible sedimentation of cells from the top of the gradient down to the bottom of the tube, the spores will not be pure.
(6) Carefully remove tubes from the centrifuge and place on ice.
(7) Collect spores from the bottom of the gradient:
  - Open the lid of the 1.5 mL gradient tube. Sterilize the bottom with 70% Ethanol.
  - Use a 23-gauge needle to **carefully and gently** drill a hole in the bottom of the tube.
  - Immediately collect the first 50–100 μL that drips out in a sterile 1.5 mL microfuge tube (2–3 drops total).
(8) Fill the microfuge tube to 1.5 mL with 1× PBS, pipetting up and down to disperse the spores. Vortex to ensure spores are evenly suspended in solution. Centrifuge for 5 min at 2000 RPM (~370× *g*). Discard the top 1.3 mL of PBS, careful not to disturb the spores at the bottom. There may not be a pellet after the first two spins.
(9) Repeat step 8.
(10) Carry out a final wash with 1.5 mL PBS and centrifuge for 1 min at maximum speed (13,200 RPM/16,100× *g*). Discard supernatant and resuspend spore pellet in ~50 µL PBS per 5 spots purified (can be adjusted according to size of spore pellet).
(11) Count spores using a hemocytometer to determine final concentration and purity. Spore yield should be ~1 × 10^6^ per 30 spots.

## 3. Quantitative Germination Assays (QGAs)

While spores play critical roles in the dispersal and survival of *Cryptococcus*, they are a largely dormant cell type that is incapable of actively replicating. To grow vegetatively and survive in new environments, including the mammalian host, spores must differentiate into yeast through the process of germination. Fungal spore germination has been studied to some degree in model systems like *Saccharomyces* and *Aspergillus*; however, the process is surprisingly poorly understood relative to other fungal processes. This is due, in part, to a lack of tools to assess germination specifically.

Traditionally, germination in most systems has been evaluated through the ability of a spore to form a colony under nutrient-rich growth conditions. This method resulted in a largely binary readout (growth/no-growth) that assessed both germination and subsequent vegetative growth. The purification of *Cryptococcus* spores en masse, along with the discoveries that germination in *Cryptococcus* is largely synchronous, follows a reproducible pattern of morphological change, and is independent of spore concentration facilitated the development of quantitative germination assays (QGAs).

QGAs are automated, microscopy-based assessments of changes in cell size and shape that correlate with stage of germination that can be made on an individual spore basis across thousands of spores in a population [14,24]. The following protocol has been optimized for use with spores from JEC20 x JEC21 crosses but has been used successfully with spores from many strain backgrounds to determine changes in germination rates and/or population dynamics in mutants of interest, variable nutrient conditions, and in the presence of chemical inhibitors [14].

### Microscopy-Based Quantitative Germination Assay

All imaging is performed on a Ti2 Elements Nikon Microscope (Nikon, Minato City, Tokyo, Japan) with automatic stage and DS-Qi2 Monochrome Microscope Camera. A humidified stage-top incubator is required to prevent evaporation and maintain proper temperature throughout the experiment (Tokai Hit Stage Top Incubator; Model STXG-TIZWX-SET, TOKAI HIT Co., Gendoji-cho, Fujinomiya, Shizuoka, Japan). An additional heating ring should be placed around the objective to be used to prevent thermal drift.

(1) The microscope and stage-top incubator need to be preheated to minimize thermal drift during long-term experiments. At least 1 h before starting the experiment, fill the stage-top incubator with dH_2_O, place the heating ring on the desired objective (20×) and turn the heating element on to 30 °C.
(2) Prepare a 384-well plate (Thermo Fisher Scientific, Waltham, MA, USA, ref no. 142762) with spores, allow them to settle, then add 2× germination medium just prior to starting the assay as follows:
  - Dilute highly pure (>97%) spores to 5000 spores/μL in 1× PBS. Add 20 μL of spores to each well (100,000 spores/well). Be sure not to touch the pipette tip to the bottom of the well as this can lead to scratches that interfere with imaging. Allow spores to settle and adhere to the bottom of the well for ~15 min at 4 °C prior to addition of media. Prepare each sample in triplicate wells.
  - Add 20 μL 2× germination media to each well, pipetting up and down gently 5 times to mix. Synthetic Medium with dextrose (2× Synthetic Minimal Medium, 4% Dextrose) is a defined medium that promotes synchronous and efficient germination.
  - Cover plate with plastic wrap to prevent evaporation during the experiment. Place the plastic plate cover on top of the plastic.
  - Place the 384-well plate in the stage-top incubator, ensuring it is pushed fully down and sitting flat on the microscope stage.
(3) Using the 20× objective (Nikon CFI Plan Apochromat Lambda 20XC), maneuver to the location of the first well. Make sure to enable the perfect focus (PFS) on the microscope. Spores are in focus when they are dark ovals without any halo-ed edges. Select X, Y, and Z coordinates for each position. Make sure that there is no overlap between images. Select three images per well for a total of nine images per sample.
(4) Once all image locations have been selected, inspect each image individually, and reset the Z coordinate so that each image is in focus, as thermal drift may occur as the experiment is being set up.
(5) Set up Z-stack imaging ranging from +7.5 μm to −7.5 μm from your selected Z-coordinates, with imaging every 1.5 μm for a total of 21 images per position. Z-stacks ensure that in-focus images will be obtained across the time course of germination, regardless of any thermal drift. This step is essential because the PFS is not designed to focus on many small cells, as is done in this assay.
(6) Set the microscope to take images every 2 h for 16 h for a total of 9 timepoints. This is the standard imaging time frame for wild type spore germination in SD medium, although longer time courses and alternative intervals can be performed as needed for individual experiments.
(7) Ensure all desired parameters are properly set for the automated experiment (time loop, each XYZ positions, Z-stack, etc.) before starting the image acquisition. Initiate image acquisition.
(8) Once the image collection is completed, export the images to TIFF files.
(9) Select the image from the Z-stack that is most in-focus for each position and time point. Spores should be dark (black/gray) and free of haloed edges.
(10) Place images in corresponding folders with the following progression/experiment/sample/time (0H, 2H, etc.)/Input. Create an “output” and “data” folder under each time point before running ImageJ.
(11) Run each sample folder through the ImageJ program (File S1). This program quantifies the area and aspect ratio of each spot in the image and compiles them into a spreadsheet.
(12) Run each sample folder through the MATLAB program (File S2). This program determines how many spots fit “spore,” “yeast,” or “other” parameters based on size and aspect ratio. It outputs a 2D histogram with size on the *X*-axis and aspect ratio on the *Y*-axis for each time point. Population level changes can be tracked over time.
(13) This program also outputs a data.csv file, which can be used for further data manipulation (creating bar plots and rate curves).

Notes: other microscopes, cameras, and temperature regulators can be used as long as the combination of lens and camera provide sufficient resolution (pixels/μm) to image cells.

It is critical that spores used in this assay are pure (<3% yeast). The presence of even a small number of yeast will result in actively replicating yeast overtaking the well during spore germination, leading to uninterpretable results.

Take care during purification and plate loading to not scrape the sides of microfuge tubes or plates. Doing so may cause debris in the samples or scratches on the plate, which obstruct the images and lead to data loss. When loading 384-well plates, avoid using the outer-most wells around the edges because they are often not as flat as those in the middle, and may create issues during imaging. Starting with well F8 is recommended. Similarly, images should be taken away from the edges of wells (~1/4 of a frame away from the edge) due to curvature that can occur in the wells of 384-well plates.

## 4. Molecular Methods

### 4.1. RNA Isolation from Spores

Spores are stress resistant particles with robust spore coats that are highly resistant to common methods for recovering RNA, such as TRIzol^®^ and bead-beating. In contrast, a very effective method for isolating RNA from spores uses hot acid phenol, which produces high quantities of total RNA with levels of purity sufficient for molecular analyses. A significant challenge when isolating RNA from spores is limiting sample; spores purified from one 15 cm V8 plate yield ~265 ng total RNA. For larger applications, such as RNA-sequencing, which can require as much as 1 µg total RNA per sample, each sample will require 4 V8 plates.

(1) Pellet purified spores by centrifuging for 5 min at 10,000 RPM (~9200× *g*) at 4 °C.
(2) Discard supernatant and resuspend the pellet in 1 mL ice-cold water. Transfer to a clean microfuge. Centrifuge at 13,200 RPM/16,100× *g* for 2 min at 4 °C and remove supernatant.
(3) Resuspend pellet in 0.4 mL TES buffer (50 mM Tris-HCl pH 7.5, 10 mM EDTA, 1% SDS). Add 0.4 mL 65 °C acid phenol:chloroform (Fisher Scientific, Hampton, NH, USA, ref no. AM9720) and vortex vigorously 10 for seconds. Incubate for 60 min at 65 °C with brief mixing every 10 min (do not vortex).
(4) Place on ice for 5 min. Centrifuge 5 min at 13,200 RPM/16,100× *g* at 4 °C.
(5) Transfer the top aqueous phase to a clean microfuge tube, add 0.4 mL chloroform, and vortex vigorously. Place on ice for 5 min and centrifuge at maximum speed at 4 °C.
(6) Transfer aqueous phase to a clean microfuge tube and add 0.4 mL chloroform. Vortex vigorously and centrifuge 5 min at maximum speed at 4 °C.
(7) Transfer aqueous phase to a new tube and precipitate the RNA by adding 0.1× volume of 3 M sodium acetate pH 5.3 (Fisher Scientific, Hampton, NH, USA ref no. R1181) (i.e., 40 μL for 400 μL aqueous phase) and 2.5× volume of ice-cold 100% ethanol. For very small amounts of RNA, add 20 μg of RNase-free glycogen (Thermo Fisher Scientific, Waltham, MA, USA, ref no. R0551) to facilitate precipitation.
(8) Centrifuge 30 min at 13,200 RPM/16,100× *g* at 4 °C to pellet precipitating RNA. Remove supernatant. Wash RNA pellet by vortexing briefly in 0.5 mL ice-cold 70% ethanol. Centrifuge 5 min at top speed at 4 °C.
(9) Remove ethanol from the pellet and evaporate remaining ethanol from the pellet. Do not over dry the pellet. EITHER:
  Place tubes upside down and allow ethanol to drain for 20 min, OR
  Place open tubes in 65 °C heat block or water bath for 10 min.
(10) Resuspend pellet in 30–50 μL RNase-free water. Vortex vigorously to ensure that the colorless pellet is fully resuspended. Determine the RNA concentration and purity spectrophotometrically by measuring the A_260_ and A_280_. Store at −80 °C or at −20 °C, if it is to be used within 1 year.

### 4.2. Protein Isolation from Spores

*Cryptococcus* spores appear resistant to mechanical forms of cell disruption such as bead beating, and attempts to make extracts using mechanical methods have resulted in very low protein yields. In contrast, ultrasonication has been used successfully to disrupt spores for protein extraction [25]. The major challenge for protein isolation is limiting spore sample. The following protocol yields ~1 μg of protein from 1 × 10^9^ spores. For that number of spores, crosses will need to be purified from ~200 15 cm V8 plates. (For context, 1 μg of total protein extract can be easily visualized if run in one lane of a protein mini-gel stained with Coomassie blue). Spores can be purified in batches and frozen until a number sufficient for protein isolation is reached.

(1) Pellet purified spores by centrifuging for 5 min at 10,000 RPM (~9200× *g*) at 4 °C.
(2) Resuspend spore pellet in 500 μL TES buffer (50 mM Tris-HCl pH 7.5, 10 mM EDTA, 1% SDS), and sonicate with a microtip 5 times for 12 s each at power 2 and a 100% duty cycle (VWR International, Radnor, PA, USA, model no. 101-135-022). Cool on ice for 1 min between cycles to avoid overheating the sample.
(3) Add 200 μL phenol-chloroform (Fisher Scientific, Hampton, NH, USA, ref no. 0883-400ML) and mix well by vortexing. Centrifuge at maximum speed (13,200 RPM/16,100× *g*) for 2 min. Discard top aqueous layer, add 1 mL 100% ethanol, and mix well.
(4) Centrifuge at maximum speed for 2 min. Discard supernatant and wash pellet with 500 μL 100% ethanol and 500 μL acetone.
(5) Discard supernatant, and dry pellet in SpeedVac for 10–15 min. The resulting protein pellet contains ~1 µg of protein/1 × 10^9^ spores and can be resuspended in 100 µL SDS PAGE loading buffer (0.025 M Tris–HCl pH 8.3, 0.192 M glycine, 0.1% SDS) and boiled for 5 min for subsequent electrophoretic separation or undergo processing for other, non-gel-based analyses.

## 5. Host–Pathogen Interactions

### 5.1. In Vitro Spore Phagocytosis Assays

*Cryptococcus* yeast are not readily phagocytosed by macrophages and other phagocytes in the absence of opsonization; however, spores are phagocytosed readily and rapidly both in vitro and in vivo [13,19]. The molecular mechanisms by which spores are phagocytosed remain largely uncharacterized, but phagocytosis appears to be key in the process of spore pathogenesis [26]. Once inside phagocytes, spores are able to germinate, and the resulting yeast are able to survive, proliferate, and escape via phagocyte rupture or through non-lytic exocytosis to evade host immune responses [13,19,27]. Two basic methods have been used to assess association and/or phagocytosis of spores by phagocytic cells: a fluorescence-based assay and a colony forming unit assay. In both methods, the association of spores with phagocytes can be determined; however, the fluorescence-based assay must be used to distinguish spore association on the surface of phagocytes from internalization of spores by phagocytes.

#### 5.1.1. Colony Forming Unit Association Assay

(1) Seed phagocytes in a sterile µ-slide 8-well coverslip/plate (Ibidi, Fitchburg, WI, USA, ref no. 80826) at a density of 2.5 × 10^5^ cells/mL in a volume of 200 µL per well. Incubate overnight to allow for settling and adhesion.
(2) Add spores at an MOI of 10:1 and allow phagocytosis to occur for 4 h.
(3) Wash three times with 1× phosphate buffered saline (PBS) to remove spores not adhered to phagocytes.
(4) Add 300 µL 0.01% Triton X-100 to each well to lyse the phagocytes. (This concentration is known to lyse mammalian phagocytes but not affect the viability of spores.)
(5) Serially dilute the lysate by 10-fold 3 times and plate each sample on a yeast peptone dextrose (YPD) agar plate. Incubate each dilution at 30 °C for 3 days and count visible colonies. Percent phagocyte association is calculated by dividing the number of colonies by the number of spores added to the phagocytes (100 × (# CFUs from lysate/CFUs introduced to phagocytes)).

#### 5.1.2. Fluorescence-Based Internalization Assay

(1) Seed phagocytic cells in a 96-well plate with ~3 × 10^4^ cells in 100 µL RPMI + 10% fetal bovine serum (FBS). Incubate cells overnight at 37 °C in 5% CO2 to allow for cell adhesion.
(2) Pellet purified spores by centrifuging for 5 min at 10,000 RPM (~9200× *g*) at 4 °C. Remove supernatant and fix spores by incubating in 4% formaldehyde overnight at 4 °C.
(3) Wash spores with 1× phosphate buffered saline (PBS), remove supernatant. Resuspend in 1× PBS and stain with 0.1 mg/mL calcofluor white (Sigma-Aldrich, St. Louis, MO, USA, ref no. 18909-100ML-F). Incubate 5–30 min at ambient temperature in the dark. Wash with 1× PBS 3 times.
(4) Remove medium from phagocytes. Add spores at an MOI of 100:1 (3 × 10^6^ spores/well) in 100 µL fresh RPMI + FBS. Incubate 4 h at 37 °C to allow for measurable phagocytosis.
(5) Measure the fluorescence of each well with a plate reader at 347 nm to determine the amount of spore-associated fluorescence in each well.
(6) Add 100 µL 0.4% trypan blue to quench fluorescence of spores that were not phagocytosed. Measure fluorescence at 347 nm of each well with a plate reader again.
(7) Generate a 10-fold serial dilution control curve to determine the amount of fluorescence per spore for calcofluor white (total). Calculate percent phagocytosis by dividing the number of spores phagocytosed by the number of spores added to the phagocytes and multiplying by 100 (100 × (no. of spores phagocytosed/no. of spores introduced)).

### 5.2. Animal Models of Infection with Spores

Both yeast and spores have been shown to cause disease in several model organisms, including moth larvae, zebrafish larvae, and mice. Protocols for infection of larvae of the moth *Galleria mellonella* with yeast are well established, and no modifications are needed for infections with spores. When caterpillars in their final instar larval stage are injected into the hemocoel with >500 yeast or spores (KN99**a** x H99), there is little or no difference in disease outcome. Both cell types result in universal mortality by 14 days post infection [12]. Similarly, in zebrafish larvae, both yeast and spores (>150 cells/zebrafish larva inoculum) cause extended fungemia that persists in the larvae until the central nervous system is breached, leading to death. Higher resolution observations of spore infections reveal that spores, unlike yeast, can be found in endothelial cells after inoculation, suggesting that habitable environments in host organisms may differ between yeast and spores [28].

#### Murine Models

Mouse models of infection have been a mainstay of virulence analyses in *Cryptococcus* for over 70 years [29]. However, due to limitations on the numbers of spores that could be purified to homogeneity, spores could not be fully evaluated for their pathogenic potential. With the isolation of large numbers of pure spores, the reliable and well-tested murine models of cryptococcal disease could be used to test the capacity of spores to cause disease and determine the nature of spore-mediated infections. As was seen in *Galleria* and zebrafish larvae, *Cryptococcus* spores from numerous species and strains, including BT63**a** x H99α, B3502 x B3501, JEC20 x JEC21, and KN99**a** x KN99α, have been shown to cause disease in murine models via tail vein injection with disease properties largely indistinguishable from yeast [12,19,30,31].

In contrast, the mouse model of intranasal infection revealed significant differences in disease progression between yeast and spores in which spore-mediated infections generate much higher fungal burdens in the brain relative to yeast. Spores germinate efficiently in the lungs of infected mice within 18 h after infection, and are preferentially trafficked out of the lungs relative to yeast. The most striking difference in disease occurs with the B3502 and B3501 strains in which spore-infected mice develop high lung fungal burdens and fulminant CNS disease after 50 days post-infection, whereas yeast-infected mice still appear healthy and do not succumb to disease [13]. Here, we detail our protocol for intranasal instillation of spores into 6–8 week old C57BL/6 mice.

Intranasal mouse infection:

(1) Prepare spores in 1× phosphate buffered saline (PBS). Count spores using a hemacytometer and prepare appropriate samples for inoculation. An effective inoculum of spores from most strain pairs is 1 × 10^5^ to 5 × 10^5^ spores per mouse. Dilute spores in enough volume of PBS for 50 µL inoculum per mouse plus an extra 10 µL for post-infection plating to confirm CFUs introduced into the mice.
(2) Place mice in a SurgiVet chamber to anesthetize with isoflurane. Set the oxygen flow rate to 2 L/minute and the isoflurane setting to 4. Maintain anesthesia throughout inoculation procedure.
(3) Suspend up to 5 anesthetized mice by their incisors on a silk thread so that the necks are fully extended.
(4) Slowly pipette half of the 50 µL of spore suspension directly into each nostril one drop at a time, ensuring that the mice fully inhale each drop. Allow the mice to remain suspended under anesthesia for 10 min after infection.
(5) After 10 min, remove mice from anesthesia chamber. Monitor mice for 10 to 15 min after they regain consciousness to ensure full recovery.
(6) Dilute remaining 10 µL inoculum to 1 spore/µL in 1× PBS and plate 100–200 µL on a yeast peptone dextrose (YPD) agar plate. Incubate for 3 days at 30 °C. Calculate the number of CFUs of the inoculum to determine infectious dose that was administered.
(7) Continue monitoring mice twice per day for 30–100 days, depending on parental strain pair used to generate spores. Check mice for signs of disease such as labored breathing, hunching, squinting, brain swelling, or paralysis. Euthanize any animals showing signs of distress or disease immediately upon discovery.

## 6. Conclusions

Spores are an essential cell type for the survival and pathogenesis of many human fungal pathogens, including *Cryptococcus*. The methods presented here provide tools for discovering new spore biology using molecular, genetic, cell biological, and biochemical approaches. As the study of infectious spores grows, an understanding of germination and spore-mediated infections will provide valuable insights into fungal-specific processes that may lead to novel therapeutic strategies against *Cryptococcus* and other invasive fungal pathogens.

## Data Availability

Not applicable.

## Supplementary Materials

The following supporting information can be downloaded at: https://www.mdpi.com/article/10.3390/jof8010004/s1, File S1: ImageJ Code, File S2: MATLAB Code.
