# Supplementary material for: Methods for Manipulating Cryptococcus Spores
Source: J Fungi (Basel). 2021 Dec 22;8(1):4. doi: 10.3390/jof8010004 (PMC8779225; doi:10.3390/jof8010004)
Supplement: Supplementary file 1 [file jof-08-00004-s001.zip › Supplementary Material File S1.pdf]

File S1 ImageJ Code

Code used to outline individual cells in microscope images.

ImageJ 1.52q
Wayne Rasband
National Institutes of Health, USA
Java 1.8.0_172 (64-bit)

Notes:

The resulting .csv files for each image need to be manually relocated to the “Data” folder for each time point.

The images that are filtered to the “Output” folder demonstrate if the program ran correctly and should be checked before moving on to the next step.

Settings > Set Measurements

- Area
- Min & Max gray value
- Shape Descriptors
- Area fraction
- Fit ellipse

Process > Batch > Macro

```
run("8-bit");
setMinAndMax(38, 166);
call("ij.ImagePlus.setDefault16bitRange", 8);
name = getTitle;
dir = getDirectory("image");

run("8-bit");
setAutoThreshold("IsoData");
setOption("BlackBackground", false);
run("Convert to Mask");
run("Close-");
run("Fill Holes");
run("Watershed");
roiManager("Show All with labels");
roiManager("Show All");
run("Analyze Particles...", "size=15-2000 circularity=0.00-1.00 show=Outlines display exclude clear");

saveAs("Results", dir+name+"results.csv");
```
