# Supplementary material for: Methods for Manipulating Cryptococcus Spores
Source: J Fungi (Basel). 2021 Dec 22;8(1):4. doi: 10.3390/jof8010004 (PMC8779225; doi:10.3390/jof8010004)
Supplement: Supplementary file 1 [file jof-08-00004-s001.zip › Supplementary Material File S2.pdf]

File S2 MATLAB Code

Code used to quantify cell types from ImageJ and produce 2D histograms.

MathWorks MATLAB
R2020b Update 3 (9.9.0.1538559)
64-bit

Notes:
You will need to change the title to the desired histogram title where “Insert Title Here” is located (i.e. Wild Type 2% Glucose 8 Hours).

Macintosh computers create “invisible” files that may need to be removed before running this program. In terminal, navigate to the desired folder and run “find . -name '.DS_Store' -type f -delete”

Depending on the µm/pixel of the user, the multiplicative factors to convert pixels^2 to µm^2 will need to be changed for eliminating debris that is too small or too large to be characterized as a cell as well as specifications of spores, yeast, or intermediates.

The definition of size and shape (area and aspect ratio) of a spore versus yeast in this protocol has been optimized for JEC20 x JEC21 spores. Spores from other strain pairs can be more or less oval and the parameters for each cell type may need to be adjusted.

Navigate to the “Data” folder of each time point and run the following:

```
%% Loading the data
raw_data = {};
raw_data{1,9} = [];
list = dir;
for i = 1:length(list)-2
   filename = list(i+2,1).name;
   delimiter = ',';
   startRow = 2;
   formatSpec = '%f%f%f%f%f%f%f%f%f%f%f%f%[^\n\r]';
   fileID = fopen(filename,'r');
   dataArray = textscan(fileID, formatSpec, 'Delimiter', delimiter, 'HeaderLines' ,startRow-1,
'ReturnOnError', false);
   fclose(fileID);
   tem = [];
   for n = 1:12
      tem(:,n) = dataArray{1,n};
   end

   row = ceil(i/9);
   m = mod(i,9);
   if m >0
      col = m;
   else
      col = m+9;
   end
   raw_data{row,col} = tem;
```

```
    clearvars filename delimiter startRow formatSpec fileID dataArray ans;
end

%% Clean the raw data - filter out debris by circularity and solidity
clean_data = {};
clean_data{1,9} = [];
s = size(raw_data);
cells = [];

for i = 1:s(1)
    for j = 1:s(2)
        [n, m] = size(cells);
        clean = [];
        table = raw_data{i,j};
        for k = 1:length(table)
            if table(k,7) >= 0.7 && table(k,12)>=0.885 && table(k,11)>=0.4 && table(k,2)>=18 &&
table(k,2)<=182.6
                clean(end+1,:) = [table(k,2:end), k];
            end
        end
        [p g] = size(clean);
        clean_data{i,j} = clean;
        cells(1:p,m+1:m+2) = [clean(:,1),clean(:,10)];
    end
end

%% Identify 'spores' and 'yeast' in each image
s = size(clean_data);
spore_indices = {};
yeast_indices = {};
other_indices = {};
labelTables = {};
labelNames = {};
for i = 1:s(1)
    for j = 1:s(2)
        table  = clean_data{i,j};
        dim    = size(table);
        nrows  = dim(1);
        spores = 0;
        yeast  = 0;
        spore_index = [];
        yeast_index = [];
        other_index = [];
        labelTable = [];
        labelName = {};
        for k = 1:nrows
            cell_area = table(k,1);
            cell_AR = table(k,10);
            if cell_area<54.3203 && cell_AR<0.8 && cell_AR>0.4
                spore_index(end+1,:) = [table(k,end), table(k,1:11)];
                labelName{k,1} = 'spore';
            elseif cell_area>=57.2827 && cell_AR>0.8
                yeast_index(end+1,:) = [table(k,end), table(k,1:11)];
                labelName{k,1} = 'yeast';
            else
```

```
                other_index(end+1,:) = [table(k,end), table(k,1:11)];
                labelName{k,1} = 'other';
            end
            labelTable(k,2) = table(k,1);
            labelTable(k,3) = table(k,10);
        end
        labelNames{i,j} = labelName;
        labelTables{i,j} = labelTable;
        spore_indices{i,j} = spore_index;
        yeast_indices{i,j} = yeast_index;
        other_indices{i,j} = other_index;
    end
end

%% Make a 2D histogram of area v. aspect ratio
xdata = [];
ydata = [];

i = 1;
for j = 1:9
    xdata = [xdata; clean_data{i,j}(:,1).*0.1369]; %the multiplicative factor converts pixels^2 to um^2
    ydata = [ydata; clean_data{i,j}(:,10)];
end
n  = 49;
xi = linspace(0, 25, 49);
yi = linspace(0.4, 1, 49);
xr = interp1(xi, 0.5:numel(xi)-0.5, xdata, 'nearest');
yr = interp1(yi, 0.5:numel(yi)-0.5, ydata, 'nearest');
Z  = accumarray([yr xr] + 0.5, 1, [n n]);

figure(1)
hold on
surf(xi, yi, Z)
title('Insert Title Here’, 'fontsize', 20)
xlabel('Area (um^2)', 'fontsize', 18, 'color', 'k')
ylabel('Aspect Ratio (width/length)', 'fontsize', 18, 'color','k')
set(gca, 'fontsize', 14, 'color','k')
line([7.43645 7.43645], [0.4 0.8], [0 50000], 'Color', [1 1 1], 'linewidth', 1)
line([7.8420768 7.8420768], [0.8 1], [0 50000], 'Color', [1 1 1], 'linewidth', 1)
line([0 7.43645], [0.8 0.8], [0 50000], 'Color', [1 1 1], 'linewidth', 1)
line([7.8420768 25], [0.8 0.8], [0 50000], 'Color', [1 1 1], 'linewidth', 1)
set(gcf,'Color',[1,1,1])

%% Export Numerical Values
Spore_Percent = mean(stacked_norm(1,1:2)) ;
Other_Percent = mean(stacked_norm(1,3:4)) ;
Yeast_Percent = mean(stacked_norm(1,5:6)) ;

Spores = {};
[rows,columns] = size(spore_indices);
for row = 1:rows
    for column=1:columns
        [srows, scols] = size(spore_indices{row, column});
        Spores = [Spores; srows];
    end
```

```
end

Other = {};
[rows,columns] = size(other_indices);
for row = 1:rows
    for column=1:columns
        [srows, scols] = size(other_indices{row, column});
        Other = [Other; srows];
    end
end

Yeast = {};
[rows,columns] = size(yeast_indices);
for row = 1:rows
    for column=1:columns
        [srows, scols] = size(yeast_indices{row, column});
        Yeast = [Yeast; srows];
    end
end


results = [Spores, Other, Yeast];

csvwrite('results.csv', results)
```
